# Actualities and Perspectives in Neurosurgery

**Published:** 2008-02-25

**Authors:** AV Ciurea, SM Iencean, FM Brehar

**Affiliations:** * First Neurosurgical Clinic, Emergency Clinic Hospital ‘Bagdasar–Arseni’,Bucharest Romania; ** Second Neurosurgical Department, Clinical Hospital ‘Sf. Treime’,Iaşi Romania

**Keywords:** neurosurgery, stereotactic radiosurgery, endovascular embolization, robotic neurosurgery

## Abstract

In the field of neurosurgery, like in other surgical specialties, the last decades have brought major achievements. The series of revolutionary discoveries has started during the last century in the fifties, with stereotactic radiosurgery, then continued with the implementation of operative microscope (during the seventies), the endovascular embolisation in the nineties and finally with the major improvement in robotic neurosurgery and molecular neurosurgery at the beginning of this century.

The major innovation has been brought not only in the field of therapeutical measures but also in the field of neuro– imaging. Thus, the modern MRI with more than 3 Tesla, can reveal to the neurosurgeon the most intimate structures of the nervous system.

Several important areas in neurosurgery like: vascular neurosurgery, functional neurosurgery and brain tumors pathology, benefit from the modern technology and from the latest discoveries from genetic and molecular biology.

In conclusion, summarizing the discoveries of the last decade, we emphasize that the related areas like genetics, molecular biology, computer technology become more and more important in the future progress of the neurosurgery.

In the field of neurosurgery, like in other surgical specialties, the last decades have brought major achievements. One of the most important of all was the development, at the beginning of the fifties, of the stereotactic neurosurgery, by Professor Lars Leksell. This technique enabled neurosurgeons to diagnose and treat by means of focused irradiation the deep and inaccessible lesions of the brain, like brain tumors and arterio–venous malformations.

Another important step was the continuous improvement of the intraoperative microscope. Therefore, at the beginning of the seventies, the microscope has been used regularly during the delicate surgical approaches of the brain and spinal cord lesions. The new techniques described by Professor Yasargil and the new concept of the compartmentation of the subarachnoid space have brought a major contribution in the field of vascular neurosurgery. These new techniques, including the manufacturing of new types of vascular clips, improve significantly mortality and morbidity in aneurysm surgery.

However, these surgical techniques are now almost overdrawn by new minimally invasive methods. During the last decades, an innovative procedure was developed by Gugliemi and his collaborators. Using this technique, the neuroradiologist is able to introduce inside the intravascular system of the patient a specially manufactured metallic coil and place it under fluoroscopic control within the aneurysm. Once placed, using an electric pulse, the coil is detached away from the catheter and starts a process of thrombosis in the aneurysm. Over the years this technique was continuously improved and the metallic coils became more and more sophisticated, this endovascular procedure therefore replacing the surgical treatment for the aneurysms located in the vertebro–basilar circulatory system.

Even if the neurosurgical field has known during the last decades major improvements in all types of pathology, there are still numerous areas, where new therapeutical solutions need to be found. Of these, we mention only the most important ones: 1. Functional neurosurgery, 2. ‘Frameless’ stereotactic neurosurgery, 3. Robotics in neurosurgery, 4. Neuroregeneration and 5. Molecular and cellular therapies in malignant brain tumors

The major progress made in computer science led to important improvements in the field of functional neurosurgery. Thus, the implantable micro–electrodes at basal ganglia level (Fig 1) replaced the old destructive procedures and became a standard in the therapeutical algorithm of Parkinson's disease.

**Fig 1:**
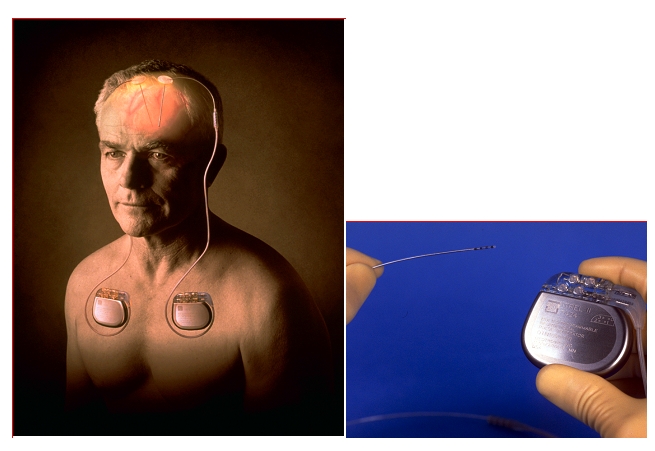
‘Deep brain stimulation’ system for bilateral stimulation of the basal ganglia in Parkinson's disease. (Medtronic.Inc)

On the other hand, the ‘deep brain stimulation’ procedure performed at globus palidum level, can be used as a therapy in other severe diseases like: generalized distonia [14] and spasmodic torticolis [12]. Some of the most surprising effects of these techniques, recently discovered during experimental stimulation, with potential benefits, are: up and down regulation of specific genes inside neurons from globus palidum [2] and enhancement of associative learning [8] .

The possibility of miniaturizing computing devices and the development, from the theoretical and technical point of view, of neural networks, enabled scientists to build artificial sensor organs like artificial retina and artificial cochlea. One of the most recent and fascinating achievements was the development of an artificial hippocampus [23]. The principles of building such a complex structure are shown in Fig 2.

**Fig 2:**
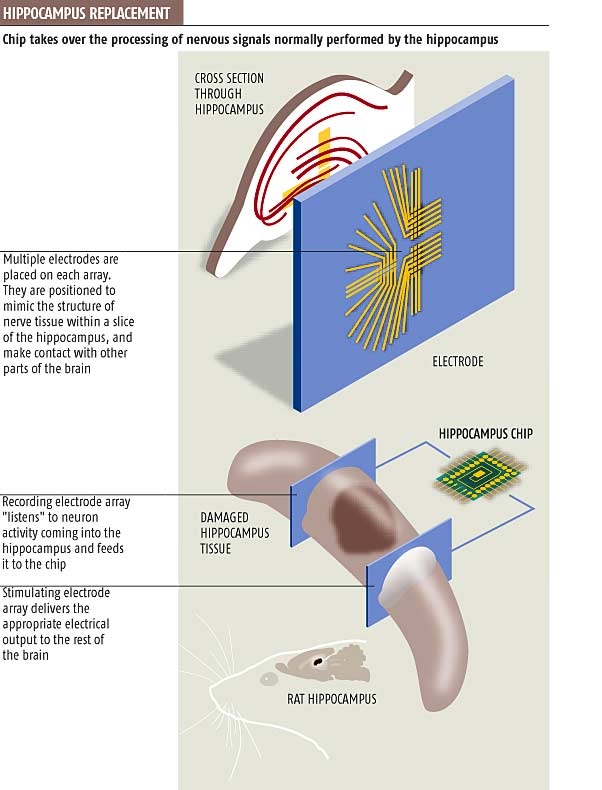
The principles of building and the functions of artificial hippocampus [Theodore Berger et al., ‘World' s first brain prosthesis revealed’, New Scientist Print Edition, 12 March 2003 (with permission)]

Stereotactic radiosurgery has known a continuous development and nowadays includes a broad field of pathology such as: deep brain tumors, malformations, cerebelo–pontine angle tumors, brain metastases, trigeminal neuralgia, involuntary movements, etc. During the last years, major improvements in neuroimaging and robotics made possible the development of frameless radiosurgery.

Neurosurgery was the first surgical specialty which took advantage of robotics, because of the precision required during the surgical procedure addressed to deep brain lesions [3, 10]. The evolution of robotics passed through several stages like: the usage of stereotactic coordinates for guiding the cerebral lesions biopsy, the acquisition of images in real time, the manufacturing of man–machine interface tools in order to improve precision of movement of the human hand (this implies also the tenfold decrease of physiological tremor) and the development of endoscopical surgical tools with remote control of movements [3, 13].

The neurosurgical robot has several basic components: the arm of the robot, the sensors located in the operation room, a six – axe moving system which controls the robot's arm, a system for locating the objects within the operation theatre in three dimensional coordinates and a computer system for data processing [3, 10, 13, 15].

The robotic systems are of three types:

- the assistive type system: the procedure is carefully planned during the preoperative period, the robot executes the pre-programmed movement and the surgeon supervises the process;
- tele–surgery: the surgeon uses a touch detection interface and performs the surgical  procedure using a special console (joystick);
- double control system: the surgeon performs the surgical procedure and the robot improves the precision of the surgeon's movement.

The Cyberknife technology is based on a combination of robotics and radiosurgery (Fig 3). This technology does not rely any more on the stereotactic frame and it has made possible the application of radiotherapy in the treatment of spinal lesions [21]. The main components of the system are two advanced computer tomographs which acquire images in real time and send this information to a powerful computer station. Using these data, the computer locates the position of the lesion in space in every moment and sends the proper command to a robotic arm which focuses a high energy X–ray beam directly to the lesion. Nowadays, this system is so precise that it can be used for the treatment of trigeminal neuralgia [16].

**Fig 3:**
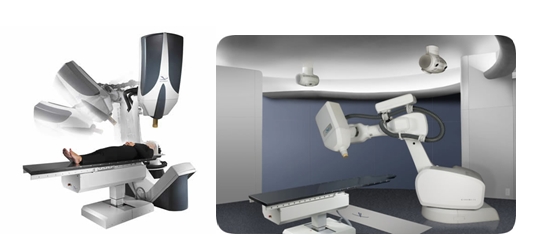
The Cyberknife system with its main components (Accuray ^TM^ Inc.)

It is well known that the nervous system can not regenerate itself because of the lack of nerve cell divisions. Therefore, many efforts have been made in the direction of neuroregeneration, using neural stem cells and even mesenchymale stem cells. The main reason of failure is the impossibility to create complex three–dimensional structures capable to function and interact with other nervous structures. We can only make such a complex structure by using new technologies like tissular engineering and nanotechnology. One of the most promising techniques is the so called MAPLE DW (matrix assisted pulsed laser evaporation direct write) (Fig 4). Using this special procedure, the cells (neural stem cells) can be seeded one by one onto a bio–degradable matrix. The matrix is specially molded in a shape similar to the structure we intend to create and contains the neurotrophic factors required for the development and differentiation of the neural stem cells into neurons [5].

**Fig 4:**
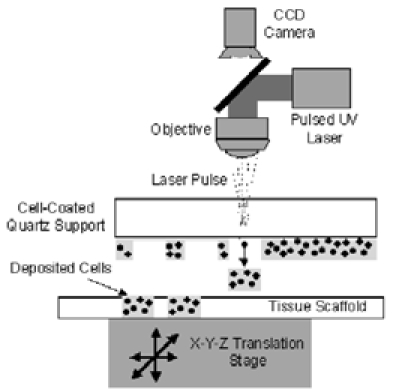
Schematic drawing of MAPLE DW (matrix assisted pulsed laser evaporation direct write)

A possible solution for spinal cord injuries could be the implant of stem cells because of their special properties of proliferation and differentiation [11, 18, and 19]. Numerous attempts have been made in this promising field with encouraging results: implants of fetal nervous tissue (Bregman,1987), implants of embryonic stem cells (Brustle, 1999), bone–marrow derived stem cells ( Prockop, 1997; Akiyama, 2002), Schwann cells (Kuhlengel, 1997) or the implant of several combinations of stem cells with a biodegradable matrix and neurotrophic molecules ( Houweling, 1998; Woerly, 2001) etc. The olfactory mucosa lines contain a special class of cells named Olfactory Ensheathing Cells (OEC). These cells have a great potential of differentiation into neurons and glial cells. Animal studies performed until now (implant with olfactory tissue – Alan MacKay–Sim, implant with olfactory glial fetal cells – Hongyuan Huang, China) proved the therapeutical potential of these cells in traumatic spinal cord injuries [11, 19, 22].

The malignant brain tumor still represents a challenging field in modern neurosurgery. Even with complex strategic therapeutic management applied (neurosurgical procedure, radiotherapy, chemotherapy) the results are poor, with 100 % mortality in glioblastoma and a medium survival of 9–12 months.

One of the major discoveries during the last years is the isolation and cultivation of tumor stem cells from glioblastoma samples [25]. This type of cells represents a better experimental model, comparable to the classic glioblastoma cells lines used before (U 87, U 1242, U 251), and retains the genotypic and phenotypic characteristics of the original tumor. Another aspect to be mentioned is that these cells represent ‘a cellular pool’ of glioblastoma. They can survive during radiotherapy and chemotherapy, then proliferate and determine the re–growth of the tumor. Because they share the same properties with neural stem cells, these cells are responsible also for the infiltrative nature of the glioblastoma. All these evidences compelled scientists to develop new drugs against these cells.

Many experiments have been performed during the last decade in order to develop an antitumoral vaccine in glioblastoma. First, some specific proteins are isolated form the glioblastoma samples. Then, a special type of white cells is isolated from the blood of the patient – the dendritic cells. These cells are pulsed with protein molecules isolated before, then are periodically injected subcutaneously in order to obtain an immune response against the protein contained within tumor cells [20, 24].

Research of the last years evidenced an extremely interesting property of the neural stem cells (obtained from fetal tissue or, according to the latest researches, even from the matured nervous tissue), namely their tropism towards the cerebral malignant tumor cells (Abody and collab. 2000) [1]. Injected at the level of the cerebral parenchyma, the stem cells have the ability to ‘trace’ tumor cells, identifying as such the secondary centers and localizing themselves at the level of the said canters (Ehtesham and collab., 2003) [6,7]. Once this capacity of neural stem cells was discovered, a problem was raised concerning their use as a therapeutical technique, by transfecting them with genes that have antitumoral potential, the synthesis products of which to be released directly at the level of the metastatic insemination centre [4]. One method is represented by the neural cell transfection with adenoviral vectors that can synthesize the TRAIL tumoricide cytokine (Hao and collab.) [9]. It was proved that this substance induces selective apoptosis in transformed cells in tumors, without impeding normal glial cells. Other authors carried out the stem cell transfection with INF-beta synthesizing gene and it was noticed that these cells synthesize high levels of IFN– beta able to destroy malign glioma cells in vitro (Nakamizo and collab, 2005) [6]. Recent research revealed also the ability of stem cells extracted from bone marrow to ‘trace’ the cerebral malignant tumor cells along their route of metastatic insemination, expressing the same tropism for the malignant tumor cells as the neural stem cells (Nakamizo si colab.) [17]. Therefore, the next step will be to obtain the bone marrow during surgical procedure, followed by isolation of mesenchymale stem cells, their transfection with tumoricide genes (IFN–beta, IL–2, etc.) and finally the inoculation of transfected stem cells in the vascular system of patients.

In conclusion, summarizing the discoveries of the last decade, we emphasize that the related areas like genetics, molecular biology and computer technology become more and more important in the future progress of neurosurgery.
